# Supplementary material for: Interpretation of T cell states from single-cell transcriptomics data using reference atlases
Source: Nat Commun. 2021 May 20;12:2965. doi: 10.1038/s41467-021-23324-4 (PMC8137700; doi:10.1038/s41467-021-23324-4)
Supplement: Supplementary file 1 — Supplementary Information [file 41467_2021_23324_MOESM1_ESM.pdf]

SUPPLEMENTARY INFORMATION

Massimo Andreatta[1,2], Jesus Corria-Osorio[1], Sören Müller[3], Rafael Cubas[4], George Coukos[1] and Santiago J. Carmona[1,2*]

[1]Ludwig Institute for Cancer Research, Lausanne Branch, and Department of Oncology, CHUV and University of Lausanne, Epalinges, 1066, Switzerland. [2]Swiss Institute of Bioinformatics, Lausanne, Switzerland. [3]Department of Bioinformatics and Computational Biology, Genentech, South San Francisco, California, United States. [4]Department of Translational Oncology, Genentech, South San Francisco, California, United States

CONTENT:

- Supplementary Table 1
- Supplementary Figures 1 to 14

Supplementary Table 1: Summary of datasets included in the mouse TIL reference atlas.

Reference dataset	Isolated population	Samples	N cells	scRNA-seq technology	Accession
Carmona	CD8+ T cells	7	6218	10x 5'	GSE116390
Ekiz	T cells	2	522	10x 3'	GSE121478
Magen_dLN	CD44+ GP66:I-Ab+ CD4+ T cells (MC38-gp66 tumor-specific)	2	950	10x 3`	GSE124691
Magen_TILs	CD44+ PD-1+ CD4+ T cells	2	781	10x 3`	GSE124691
MC38_dLN	T cells (except a CD44- CD62L+ fraction to enrich for non-naive)	4	3488	10x 5'	E-MTAB-9274
Singer	CD8+ T cells	4	410	smart-seq2	GSE86028
Xiong	T cells	4	4434	10x 5'	E-MTAB-7919

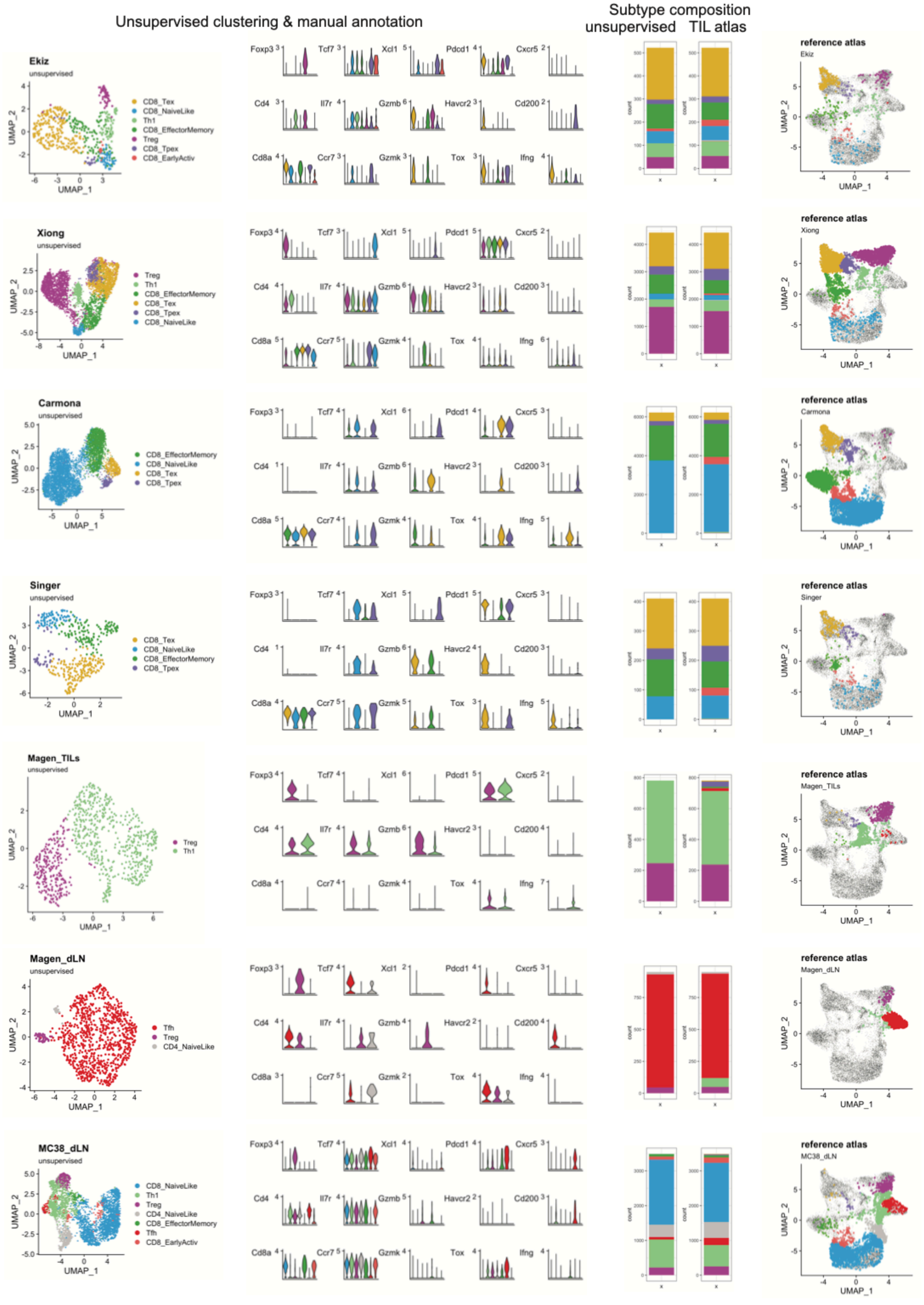

Supplementary Figure 1: Unsupervised clustering, annotation and composition of datasets included in the reference TIL atlas. For each of the seven datasets composing the reference TIL atlas, we show unsupervised clusters (left); average expression by unsupervised cluster for a panel of marker genes (center-left); subtype composition of the unsupervised clusters compared to the reference clusters (center-right); and distribution of cells on the reference map, colored by reference cluster (right). Note that unsupervised clusters utilize the same nomenclature as the reference cluster, but are determined independently and in an unsupervised manner.

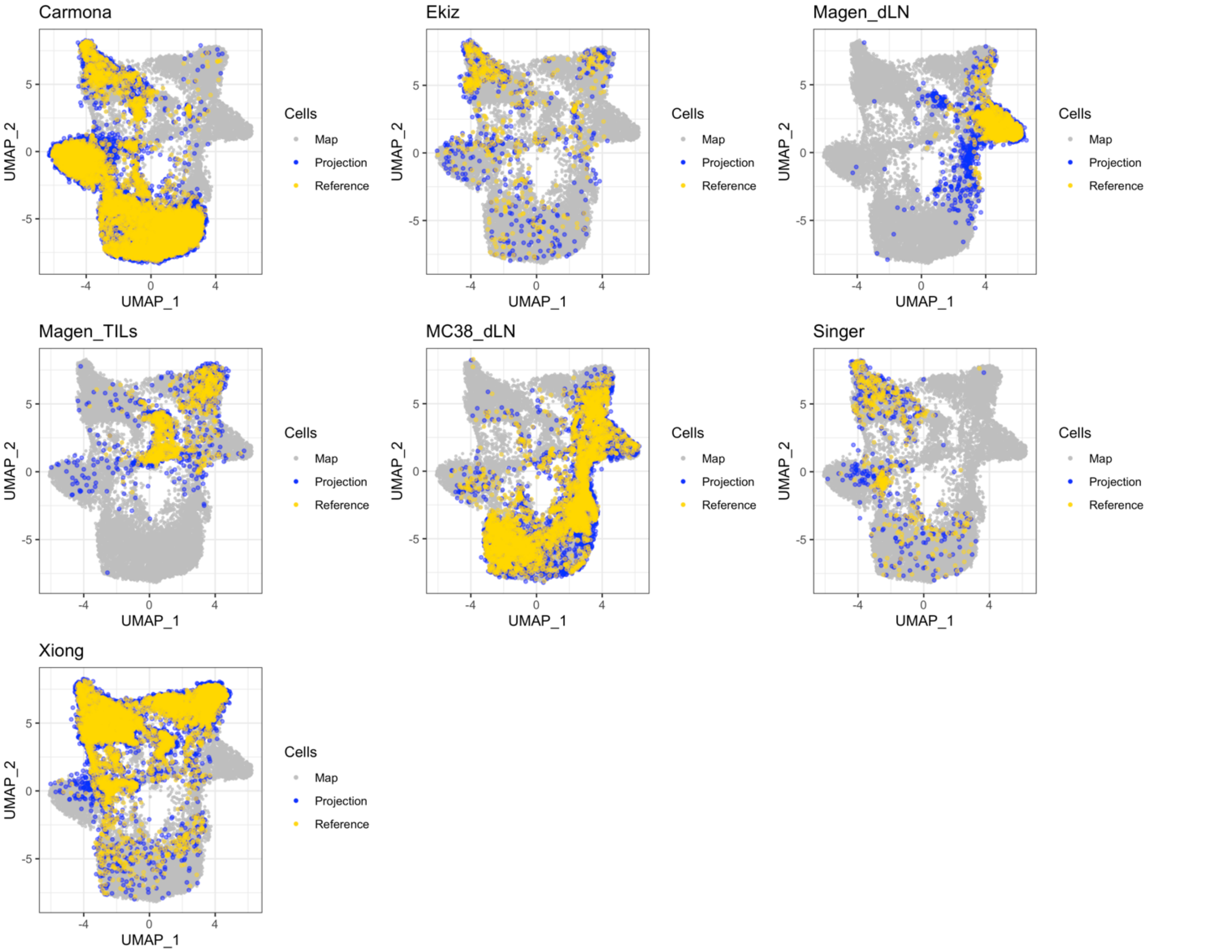

Supplementary Figure 2: Cross-validated projection of dataset in the reference map. For each of the seven datasets included in the reference TIL map, we constructed a cross-validation experiment by removing, at each cross-validation step, half of the samples of a given dataset, and then projected these samples into a reduced version of the map that does not contain the data points from these samples. The projected coordinates (blue) can be compared against the original coordinates in the map (yellow); the complete map layout is shown in gray. Note that the distribution pattern of each dataset is associated to the specific T cell subtypes it contains, e.g. datasets Carmona and Singer contain CD8 TILs, Magen_TILs contain CD4 TILs, Ekiz and Xiong contain both CD4 and CD8 TILs, Magen_dLN and MC38_dLN contain CD4 T cells, and both CD4 and CD8 T cells, respectively, from tumor-draining lymph nodes.

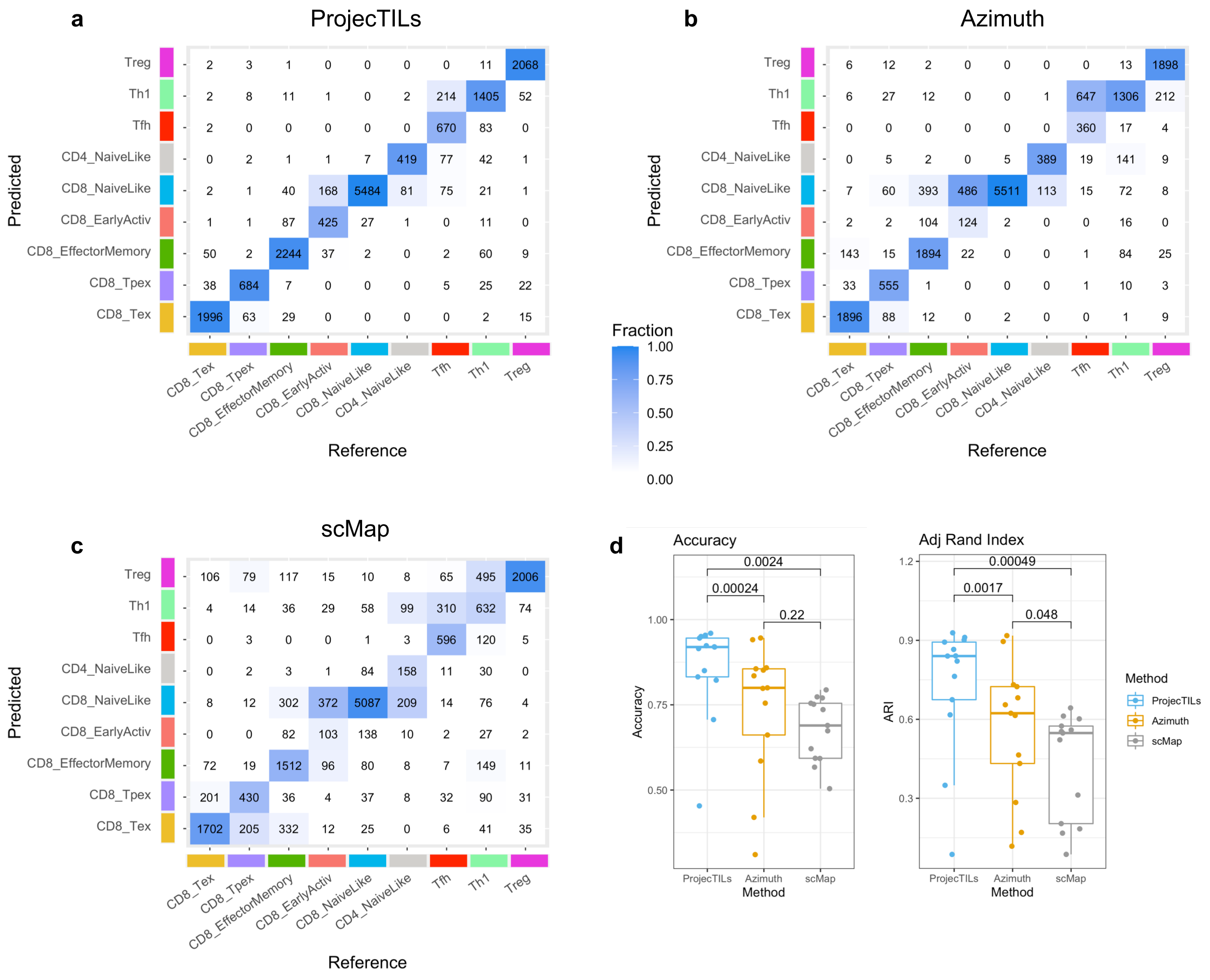

Supplementary Figure 3: Cross-validated performance for ProjecTILs, Azimuth and scmap in cell state classification. Combined confusion matrix of reference vs. predicted T cell states for the three methods ProjecTILs (**a**), Azimuth (**b**) and scmap (**c**). The fraction of correct predictions (values on the diagonal) with respect to the total number of cells represent the accuracy of the method. (**d**) Accuracy and Adjusted Rand Index (ARI) of individual cross-validated subsets (n=13) for the three methods. p-values were calculated using the two-sided Wilcoxon sign-rank test and are reported for each comparison. Boxes in box-and-whisker plots are defined by median and interquartile range (IQR); whiskers are drawn up to the largest (or smaller) observed points falling within a distance of 1.5 times the IQR measured from the hinge (median).

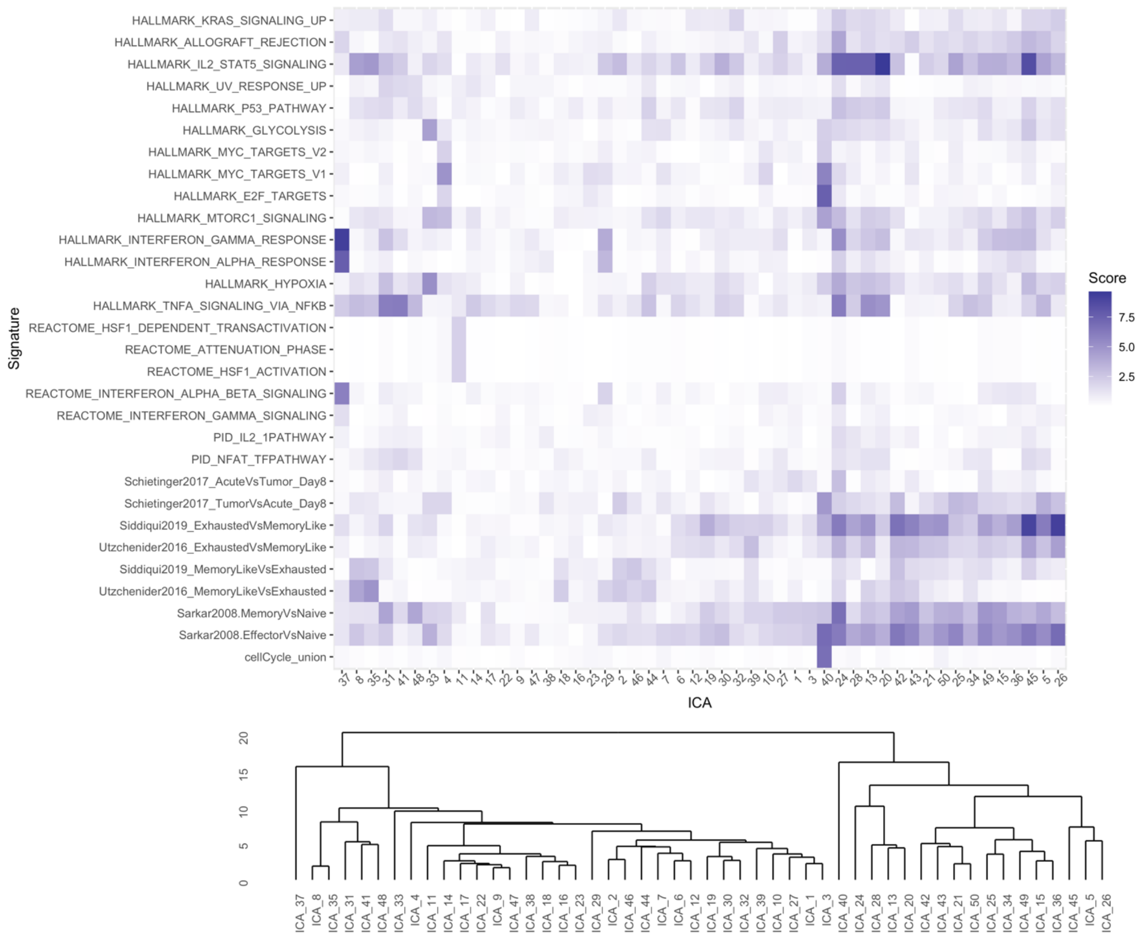

Supplementary Figure 4: Correlation between ICA genes and annotated signatures from mSigDB. Score for each ICA component against a selection of gene signatures from the Molecular Signature Database (top). Dendrogram of ICA components clustered by signature similarity (bottom).

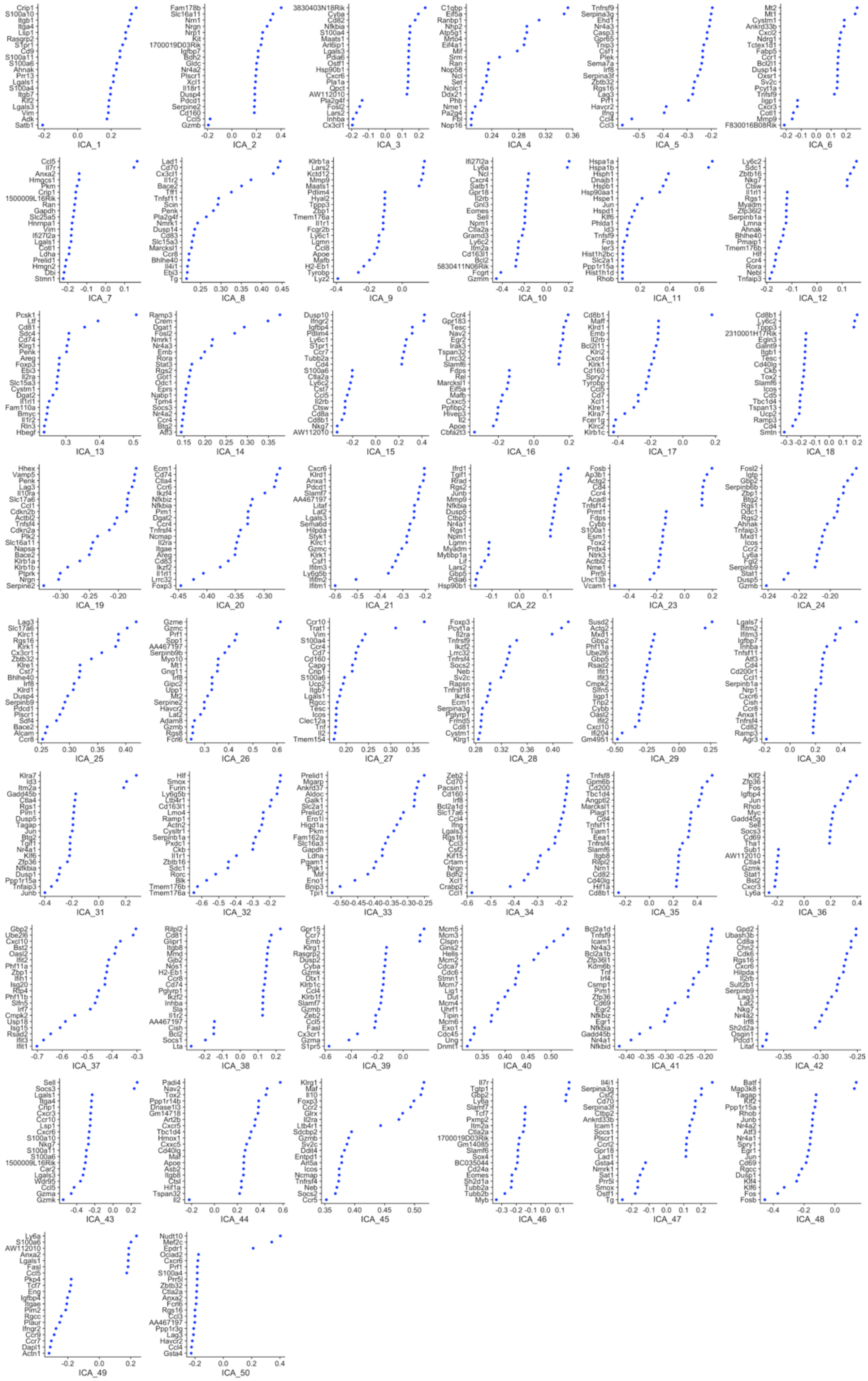

Supplementary Figure 5: Top 20 genes associated with each of the 50 ICA dimensions of the reference TIL atlas. The scale on the x-axis represents the ICA loading of each gene, and gives an indication of the contribution of each gene to the cell coordinate in the given ICA component.

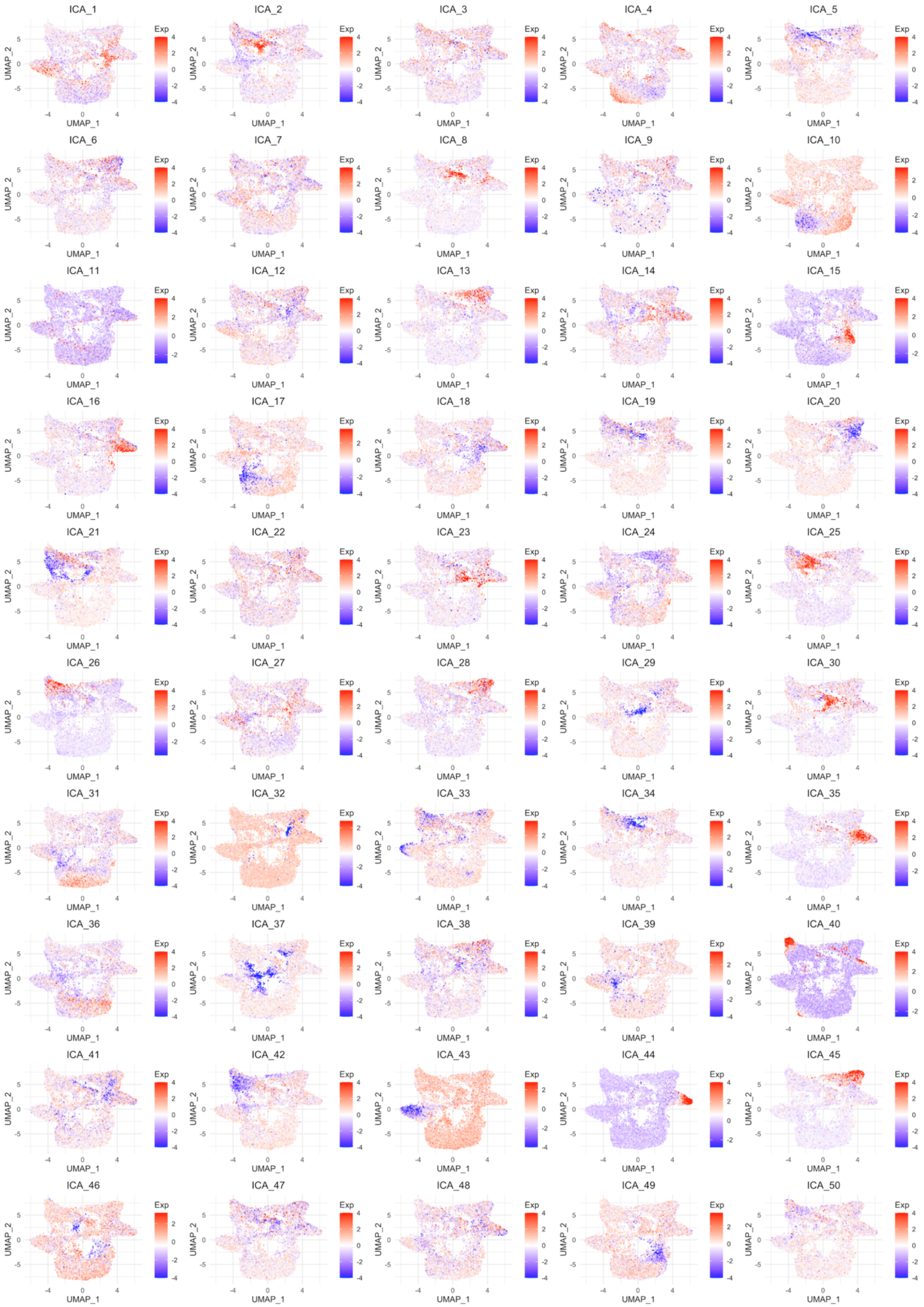

Supplementary Figure 6: Reference cell embeddings in the 50 ICA dimensions of the reference TIL atlas. The Exp scale represents the coordinate of each cell for the given ICA component.

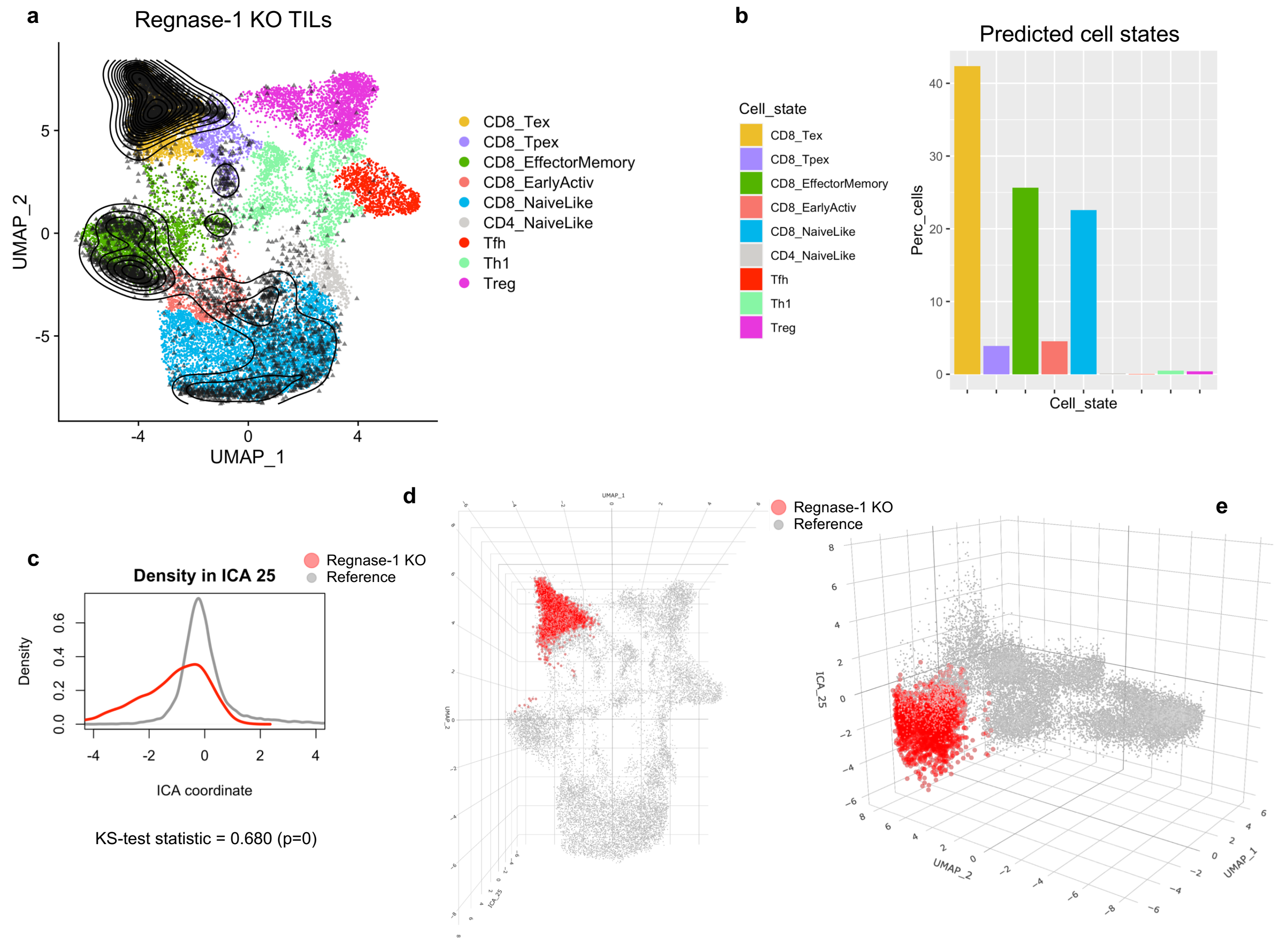

Supplementary Figure 7: a) Projection of Regnase-1 KO single-cell data onto the TIL reference atlas, without control sample. **b)** Predicted cell subtype composition in terms of nearest neighbors on the reference map. **c)** ICA component 25 was ranked as the second most significant by two-sample, two-sided KS-test (statistic=0.680, p=0) for the null hypothesis that ICA 25 embeddings for CD8 exhausted Regnase-1 KO cells and CD8 exhausted reference cells come from the same distribution. **d-e)** Top and side view of (predicted) CD8 exhausted cells from Regnase-1 KO in UMAP space, with ICA 25 embeddings on the z-axis.

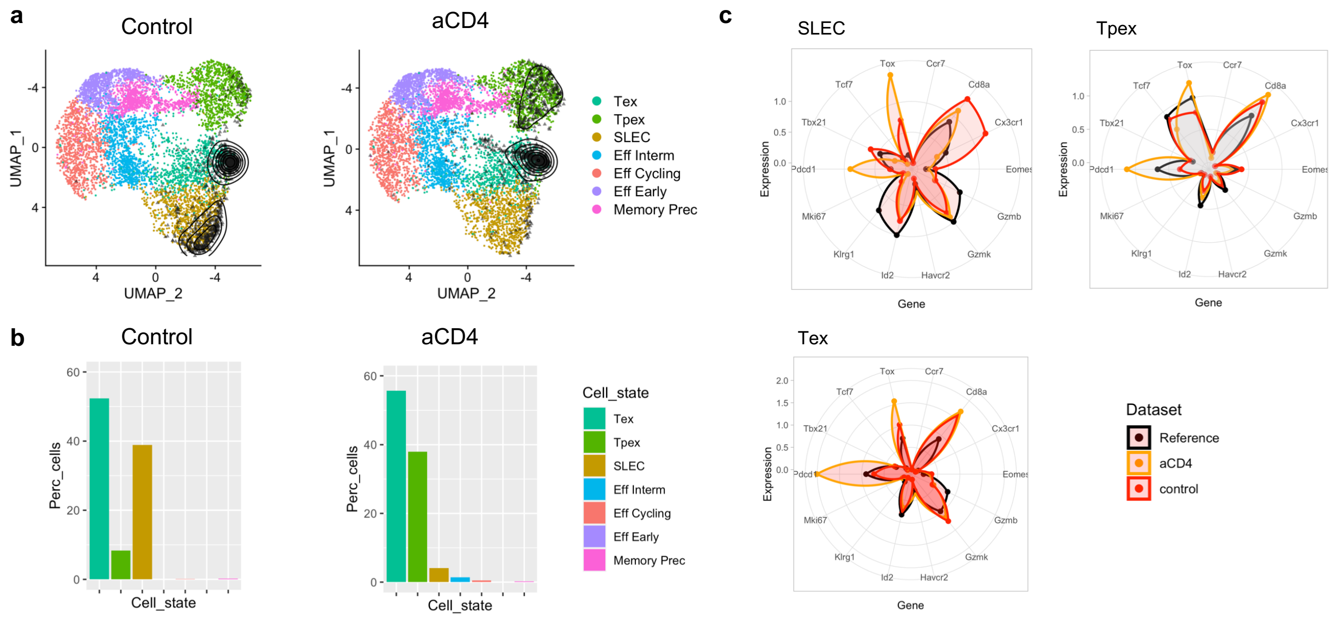

Supplementary Figure 8: a) Projection of Control and CD4-depleted (aCD4) samples from Kanev *et al.* onto the LCMV reference atlas. **b)** Predicted cell subtype composition in terms of percentage of cells (Perc_cells) for Control and aCD4 treatment, showing a shift from short-lived effectors (SLEC) to Precursor Exhausted (Tpex) phenotype upon CD4 depletion. **c)** Normalized gene expression profile for selected marker genes for the SLEC, Tpex and Tex cell types.

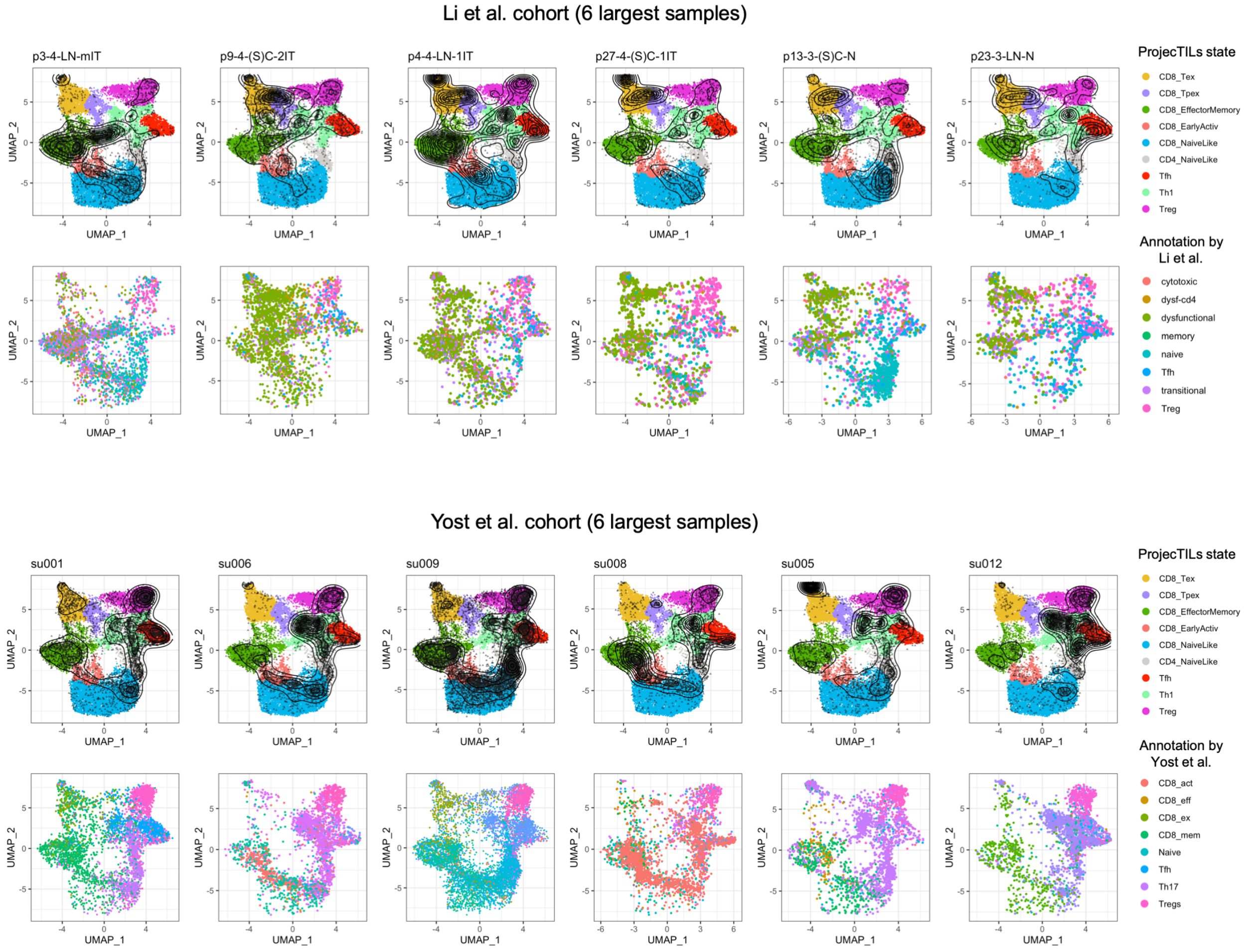

Supplementary Figure 9: Projection of human scRNA-seq data onto the reference TIL atlas for 12 individual samples in two cancer patient cohorts. For the two cohorts (Li et al. and Yost et al.), each column represents a patient of the six with the largest number of cells. The top rows display the density of projected cells (as a contour line) over the reference murine TIL atlas, the bottom rows show individual projected cells colored by the original annotation by the authors. We note that patient su008 in the Yost et al. cohort has a disproportionate amount of cells annotated by the authors as Activated T cells (CD8_act) and projected in different sectors of the reference map; indeed, this subtype appears to be defined nearly completely by patient su008 (see Yost et al. (2019) Nature Medicine, Figure 2a), suggesting that the original definition of the Activated T cell subtype is largely explained by uncorrected batch effects.

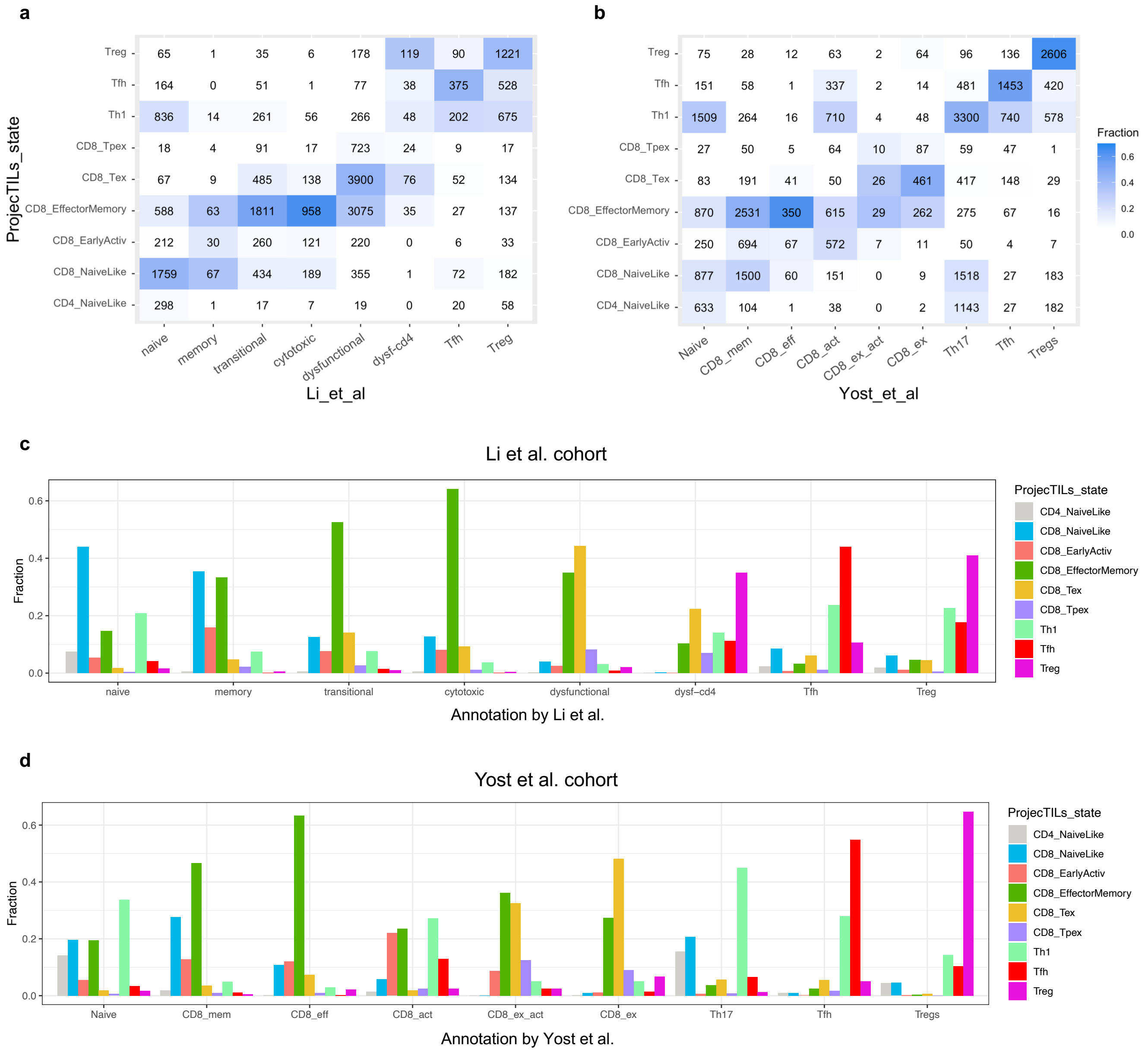

Supplementary Figure 10: Concordance of ProjecTILs classification with cell annotation from original studies. a) ProjecTILs predicted states vs. original annotation for human T cell scRNA-seq data from Li *et al.*; numbers indicate absolute amount of cells for each annotation pair, colours indicate fraction over the total number in the original study annotation. **b)** Same as previous panel, but using the data and annotations from Yost *et al.* **c)** For each T cell type defined by Li *et al.*, fraction of cell assigned by ProjecTILs to the cell states of the mouse reference TIL atlas. **d)** For each T cell type defined by Yost *et al.*, fraction of cell assigned by ProjecTILs to the cell states of the mouse reference TIL atlas.

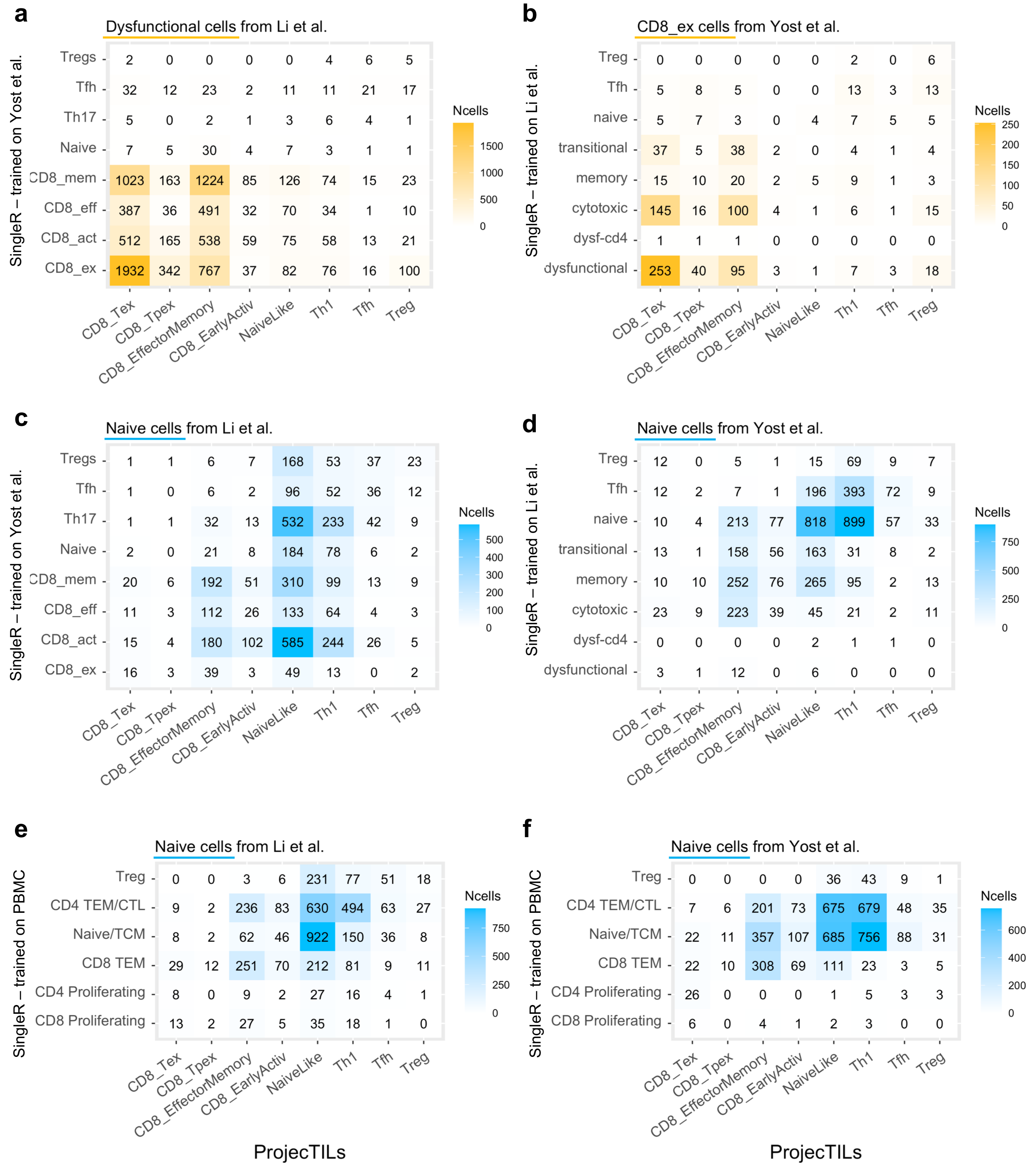

Supplementary Figure 11: Classification of human TILs from two cohorts using singleR models. a) TILs from the Li et al. cohort originally annotated as "dysfunctional" were classified using a singleR predictor trained on expression profiles from the Yost et al. cohort; singleR classification is compared to the ProjecTILs annotation for the same cells. **b)** TILs from the Yost et al. cohort originally annotated as "CD8_ex" were classified using a singleR predictor trained on expression profiles from the Li et al. cohort; singleR classification is compared to the ProjecTILs annotation for the same cells. **c-d)** Same as A-B, for cells originally annotated as "naive". **e-f)** TILs from the Li et al. cohort (**e**) and Yost et al. cohort (**f**) originally annotated as "naive" were classified using a singleR predictor trained on human PBMC expression profiles and annotations; singleR classification is compared to the ProjecTILs annotation for the same cells.

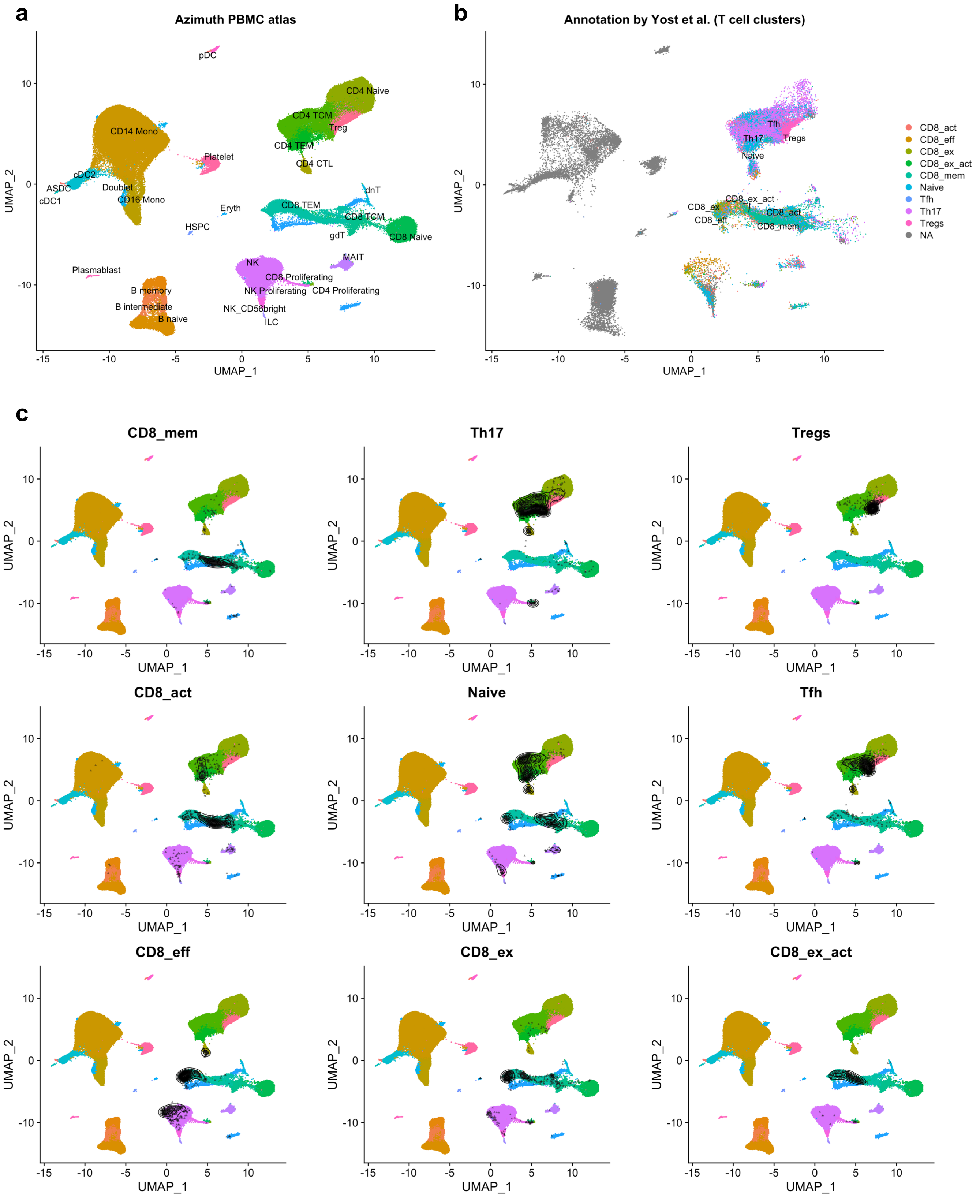

Supplementary Figure 12: Projection of single-cell data from Yost et al. cohort using Azimuth and its default PBMC reference atlas. a) PBMC reference atlas provided by Azimuth; **b**) Projected query T cell data, colored by the original annotation by Yost et al.; **c**) Each panel represents the location and density of projected T cells for one cluster annotated in the original Yost et al. study.

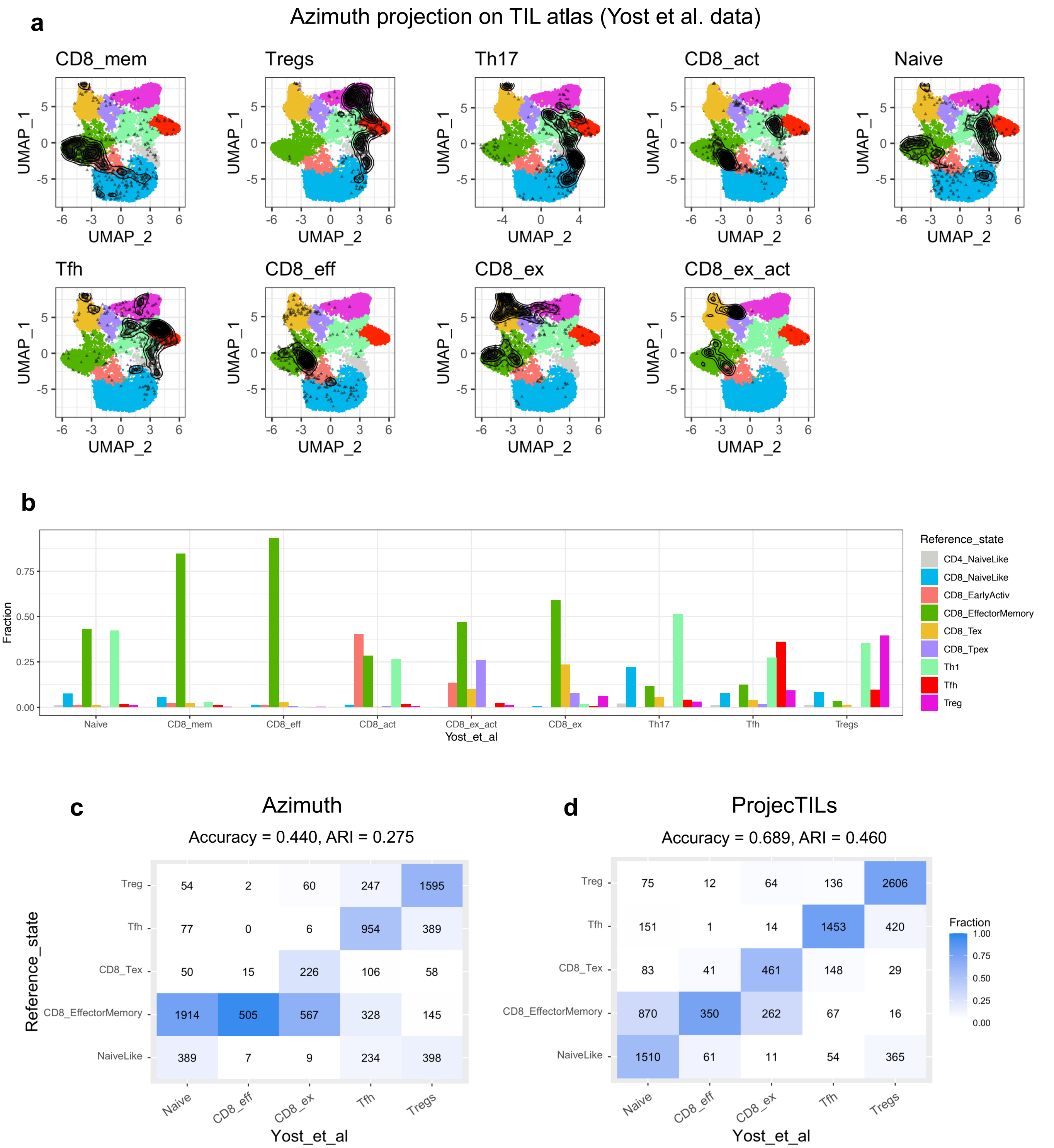

Supplementary Figure 13: Projection of single-cell data from Yost et al. cohort using Azimuth and the reference murine T cell atlas constructed in our study. a) For each of the nine T cell subtypes defined by Yost et al., individual points identify projected cells over the murine T cell atlas, and contour lines represent the density of points in a given area of the atlas. **b**) Fraction of cells for each original annotation that were assigned to the reference cell states of the reference atlas. **c-d**) Confusion matrix for Azimuth (**c**) and for ProjecTILs (**d**) for five subtypes that could be confidently matched between the original annotation and the reference atlas. For this comparison, CD4_ NaiveLike and CD8_NaiveLike were combined in a single category, NaiveLike.

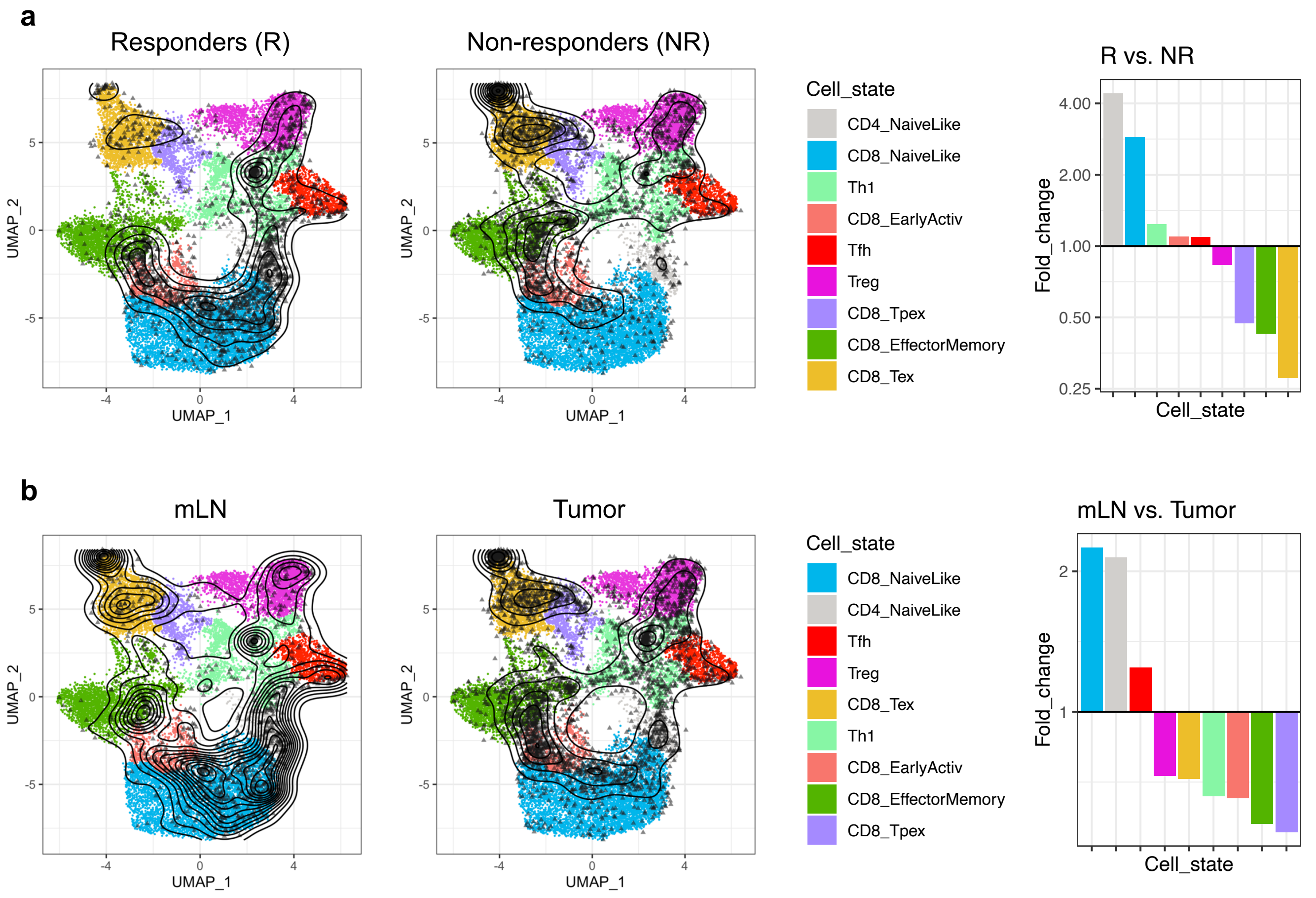

Supplementary Figure 14: ProjecTILs analysis of baseline scRNA-seq data from melanoma patients (Sade-Feldman et al.). a) Distribution of projected query cells by response to immune checkpoint blockade, and relative enrichment of reference murine TIL states in responders (R) vs. non-responders (NR). **b)** Distribution of projected query cells by biopsy site (mLN: metastatic lymphnode vs. tumor), and relative enrichment of reference murine TIL states in the two tissues.
