# Supplementary material for: Interpretation of T cell states from single-cell transcriptomics data using reference atlases
Source: Nat Commun. 2021 May 20;12:2965. doi: 10.1038/s41467-021-23324-4 (PMC8137700; doi:10.1038/s41467-021-23324-4)
Supplement: Supplementary file 3 — Description of Additional Supplementary Files [file 41467_2021_23324_MOESM3_ESM.pdf]

**Description of Additional Supplementary Files**

File Name: Supplementary Data 1
Description: Average gene expression profiles for the nine T cell subtypes included in the reference TIL atlas for mouse (TIL_atlas_profiles sheet) and for human (TIL_atlas_profile_human sheet). For human profiles, only genes with a clear mouse ortholog are included. Cell subtype gene signatures for mouse (Mouse_signatures sheet) and human (Human_signatures sheet) derived from the reference TIL atlas, including at most 25 genes per signature.
