# Supplementary material for: Interpretation of T cell states from single-cell transcriptomics data using reference atlases
Source: Nat Commun. 2021 May 20;12:2965. doi: 10.1038/s41467-021-23324-4 (PMC8137700; doi:10.1038/s41467-021-23324-4)
Supplement: Supplementary file 5 — Reporting Summary [file 41467_2021_23324_MOESM5_ESM.pdf]

nature research

Corresponding author(s): Santiago J. Carmona
Last updated by author(s): Mar 16, 2021

Reporting Summary

Nature Research wishes to improve the reproducibility of the work that we publish. This form provides structure for consistency and transparency in reporting. For further information on Nature Research policies, see our Editorial Policies and the Editorial Policy Checklist.

Statistics

For all statistical analyses, confirm that the following items are present in the figure legend, table legend, main text, or Methods section.

n/a	Confirmed	
☐	☒	The exact sample size (*n*) for each experimental group/condition, given as a discrete number and unit of measurement
☐	☒	A statement on whether measurements were taken from distinct samples or whether the same sample was measured repeatedly
☐	☒	The statistical test(s) used AND whether they are one- or two-sided<br>*Only common tests should be described solely by name; describe more complex techniques in the Methods section.*
☐	☒	A description of all covariates tested
☐	☒	A description of any assumptions or corrections, such as tests of normality and adjustment for multiple comparisons
☒	☐	A full description of the statistical parameters including central tendency (e.g. means) or other basic estimates (e.g. regression coefficient) AND variation (e.g. standard deviation) or associated estimates of uncertainty (e.g. confidence intervals)
☐	☒	For null hypothesis testing, the test statistic (e.g. *F*, *t*, *r*) with confidence intervals, effect sizes, degrees of freedom and *P* value noted<br>*Give P values as exact values whenever suitable.*
☒	☐	For Bayesian analysis, information on the choice of priors and Markov chain Monte Carlo settings
☒	☐	For hierarchical and complex designs, identification of the appropriate level for tests and full reporting of outcomes
☒	☐	Estimates of effect sizes (e.g. Cohen's *d*, Pearson's *r*), indicating how they were calculated

Our web collection on statistics for biologists contains articles on many of the points above.

Software and code

Policy information about availability of computer code

Data collection	Data were downloaded from NCBI GEO or EBI Arrayexpress repositories, accession codes in the manuscript
Data analysis	Code used in this study: ProjecTILs v0.6.1 [https://github.com/carmonalab/ProjecTILs], STACAS v1.0.1 [https://github.com/carmonalab/STACAS], Azimuth/Seurat v4 [https://github.com/satijalab/seurat], Seurat v3.2.3 [https://github.com/satijalab/seurat], TILPRED v1.0 [https://github.com/carmonalab/TILPRED], scmap v1.12.0 [https://github.com/hemberg-lab/scmap], SingleR v1.2.4 [https://bioconductor.org/packages/release/bioc/html/SingleR.html], scRepertoire v1.0.0 [https://github.com/ncborcherding/scRepertoire]. Code to reproduce ProjecTILs data analysis available at https://carmonalab.github.io/ProjecTILs_CaseStudies/

For manuscripts utilizing custom algorithms or software that are central to the research but not yet described in published literature, software must be made available to editors and reviewers. We strongly encourage code deposition in a community repository (e.g. GitHub). See the Nature Research guidelines for submitting code & software for further information.

Data

Policy information about availability of data

All manuscripts must include a data availability statement. This statement should provide the following information, where applicable:
- Accession codes, unique identifiers, or web links for publicly available datasets
- A list of figures that have associated raw data
- A description of any restrictions on data availability

Generated scRNA-seq data of MC38 tumor-draining lymph node T cells were deposited in the ArrayExpress database with accession ID E-MTAB-9274 [https://www.ebi.ac.uk/arrayexpress/experiments/E-MTAB-9274/]. To construct the reference TIL atlas, we obtained single-cell gene expression matrices from the following GEO entries: GSE124691 [https://www.ncbi.nlm.nih.gov/geo/query/acc.cgi?acc=GSE124691]25, GSE116390 [https://www.ncbi.nlm.nih.gov/geo/query/acc.cgi?acc=GSE116390], GSE121478 [https://www.ncbi.nlm.nih.gov/geo/query/acc.cgi?acc=GSE121478], GSE86028 [https://www.ncbi.nlm.nih.gov/geo/query/acc.cgi?

acc=GSE86028]; and entry E-MTAB-7919 [https://www.ebi.ac.uk/arrayexpress/experiments/E-MTAB-7919]71 from Array-Express. For the TIL projection examples (OVA Tet+, miR-155 KO and Regnase-KO), we obtained the gene expression counts from entries GSE122713 [https://www.ncbi.nlm.nih.gov/geo/query/acc.cgi?acc=GSE122713], GSE121478 [https://www.ncbi.nlm.nih.gov/geo/query/acc.cgi?acc=GSE121478] and GSE137015 [https://www.ncbi.nlm.nih.gov/geo/query/acc.cgi?acc=GSE137015], respectively.
Single-cell data to build the LCMV-specific CD8 T cell reference map were downloaded from GEO under the following entries: GSE131535 [https://www.ncbi.nlm.nih.gov/geo/query/acc.cgi?acc=GSE131535], GSE134139 [https://www.ncbi.nlm.nih.gov/geo/query/acc.cgi?acc=GSE134139] and GSE119943 [https://www.ncbi.nlm.nih.gov/geo/query/acc.cgi?acc=GSE119943], selecting only samples in wild type conditions. Data for the Ptpn2-KO, Tox-KO and CD4-depletion projections were obtained from entries GSE134139 [https://www.ncbi.nlm.nih.gov/geo/query/acc.cgi?acc=GSE134139], GSE119943 [https://www.ncbi.nlm.nih.gov/geo/query/acc.cgi?acc=GSE119943], and GSE137007 [https://www.ncbi.nlm.nih.gov/geo/query/acc.cgi?acc=GSE137007] and were not included in the construction of the reference map. Single-cell expression matrices for LCMV-specific CD8 T cells in multiple tissues (Figure 5) were kindly provided by the authors47; raw single-cell data are also available at ENA under accession code PRJEB36998 [https://www.ebi.ac.uk/ena/browser/view/PRJEB36998].
Processed single-cell RNA-seq gene expression matrices from cancer patient samples were downloaded from GEO under the following entries: GSE123139 (Melanoma_Li) [https://www.ncbi.nlm.nih.gov/geo/query/acc.cgi?acc= GSE123139], GSE123813 (BasalCC_Yost) [https://www.ncbi.nlm.nih.gov/geo/query/acc.cgi?acc=GSE123813], GSE120575 (Melanoma_Sade-Feldman) [https://www.ncbi.nlm.nih.gov/geo/query/acc.cgi?acc=GSE120575], GSE115978 (Melanoma_Jerby-Arnon) [https://www.ncbi.nlm.nih.gov/geo/query/acc.cgi?acc=GSE115978], GSE114727 (Breast_Azizi) [https://www.ncbi.nlm.nih.gov/geo/query/acc.cgi?acc=GSE114727]. For the liver, lung and colorectal cancer samples, we used the single-cell expression matrices collected by Nieto et al. [https://doi.org/10.5281/zenodo.4263972].
Source data for the TIL and viral reference atlases were deposited in figshare with DOI 10.6084/m9.figshare.12478571 [https://doi.org/10.6084/m9.figshare.12478571] and 10.6084/m9.figshare.12489518 [https://doi.org/10.6084/m9.figshare.12489518], respectively.

Field-specific reporting

Please select the one below that is the best fit for your research. If you are not sure, read the appropriate sections before making your selection.

- [x] Life sciences
- [ ] Behavioural & social sciences
- [ ] Ecological, evolutionary & environmental sciences

For a reference copy of the document with all sections, see nature.com/documents/nr-reporting-summary-flat.pdf

Life sciences study design

All studies must disclose on these points even when the disclosure is negative.

Sample size	To explore T cell diversity in mice tumor-draining lymph nodes, 4 samples/biological replicates were collected for scRNA-seq analysis. For scRNA-seq analysis this sample size is considered large due to their high cost.
Data exclusions	low-quality single-cell transcriptomes were excluded from analysis using standard criteria described in methods
Replication	T cell diversity was reproducible across all 4 samples/biological replicates in scRNA-seq analysis.
Randomization	Not relevant. This study did not involve experimental grouping
Blinding	Not relevant. This study did not involve experimental grouping

Reporting for specific materials, systems and methods

We require information from authors about some types of materials, experimental systems and methods used in many studies. Here, indicate whether each material, system or method listed is relevant to your study. If you are not sure if a list item applies to your research, read the appropriate section before selecting a response.

Materials & experimental systems

n/a	Involved in the study	
☐	☒	Antibodies
☐	☒	Eukaryotic cell lines
☒	☐	Palaeontology and archaeology
☐	☒	Animals and other organisms
☐	☒	Human research participants
☒	☐	Clinical data
☒	☐	Dual use research of concern

Methods

n/a	Involved in the study	
☒	☐	ChIP-seq
☒	☐	Flow cytometry
☒	☐	MRI-based neuroimaging

April 2020

Antibodies

Antibodies used	anti CD45 (clone 30-F11, 1:100 Cat. # 103116) and TCRb (clone H57-597, 1:100 Cat. # 109208) purchased from BioLegend, CD62L (clone Mel14, 1:80 Cat. # 48-0621-82), and CD44 (clones IM7, 1:200 Cat. # 17-0441-82) from eBioscience. LIVE/DEAD Fixable Dead Cell Stain from Life Technologies (Cat. # L34966) was used to gate on live cells.
Validation	All used antibodies have been validated for their use in flow cytometry of mouse lymphocytes

Eukaryotic cell lines

Policy information about cell lines

Cell line source(s)	The murine colon adenocarcinoma MC38 cell line was obtained from a former Genentech colleague, Rink Offringa, in 2008 (original source unknown)
Authentication	MC38 cell line was not authenticated in the past year
Mycoplasma contamination	MC38 cell line was not tested for mycoplasma contamination
Commonly misidentified lines (See ICLAC register)	none

Animals and other organisms

Policy information about studies involving animals; ARRIVE guidelines recommended for reporting animal research

Laboratory animals	Eight- to 10-week-old female C57Bl/6 mice were obtained from The Charles River Laboratories and housed at Genentech in standard rodent micro-isolator cages
Wild animals	The study did not involve wild animals
Field-collected samples	The study did not involve field-collected samples
Ethics oversight	All animal studies were reviewed and approved by Genentech's Institutional Animal Care and Use Committee. Mice whose tumors exceeded acceptable size limits (2,000 mm3) or became ulcerated were euthanized and removed from the study.

Note that full information on the approval of the study protocol must also be provided in the manuscript.

Human research participants

Policy information about studies involving human research participants

Population characteristics	Human data analyzed in this manuscript were obtained from previous studies, see Data Availability for more information.
Recruitment	Human data analyzed in this manuscript were obtained from previous studies, see Data Availability for more information.
Ethics oversight	Human data analyzed in this manuscript were obtained from previous studies, see Data Availability for more information.

Note that full information on the approval of the study protocol must also be provided in the manuscript.
